# Prediction of radiotherapy response with a 5‐microRNA signature‐based nomogram in head and neck squamous cell carcinoma

**DOI:** 10.1002/cam4.1369

**Published:** 2018-02-23

**Authors:** Lin Chen, Yihui Wen, Jingwei Zhang, Wei Sun, Vivian W. Y. Lui, Yi Wei, Fenghong Chen, Weiping Wen

**Affiliations:** ^1^ Department of Otolaryngology the First Affiliated Hospital of Sun Yat‐sen University Guangzhou China; ^2^ Guangzhou Key Laboratory of Otorhinolaryngology Guangzhou China; ^3^ Department of Medical Ultrasonics Institute of Diagnostic and Interventional Ultrasound the First Affiliated Hospital of Sun Yat‐sen University Guangzhou China; ^4^ Faculty of Medicine School of Biomedical Sciences the Chinese University of Hong Kong Hong Kong SAR

**Keywords:** Head and neck cancer, microRNA, nomograms, radiotherapy

## Abstract

Radiotherapy is unlikely to benefit all patients with head and neck squamous cell carcinoma (HNSCC). Therefore, novel method is warranted to predict the radiotherapy response. Our study aimed to construct a microRNA (miRNA)‐based nomogram to predict clinical outcomes of patients with HNSCC receiving radiotherapy. We screened out 56 differential miRNAs by analyzing 44 paired tumor and adjacent normal samples miRNA expression profiles from The Cancer Genome Atlas (TCGA). A total of 307 patients with HNSCC receiving adjuvant radiotherapy were randomly divided into a training set (*n* = 154) and a validation set (*n* = 153). In the training set, we combined the differential miRNA profiles with clinical outcomes, and LASSO regression model was applied to establish a 5‐miRNA signature. The prediction accuracy of the 5‐miRNA signature was further validated. In addition, target genes of these miRNAs were predicted, and Gene Ontology (GO) analysis as well as KEGG pathway analysis was executed. A 5‐miRNA signature including miR‐99a, miR‐31, miR‐410, miR‐424, and miR‐495 was identified. With a cutoff value of 1.2201 from Youden's index, the training set was divided into high‐risk and low‐risk groups, and the 5‐year overall survival was significantly different (30% vs. 73%, HR 3.65, CI 2.46–8.16; *P *<* *0.0001). Furthermore, our 5‐miRNA signature revealed that only low‐risk group would benefit from radiotherapy. Then, a nomogram combining 5‐miRNA signature with clinical variables to predict radiotherapy response was constructed. The analysis of 108 target genes of these miRNAs revealed some potential mechanisms in HNSCC radiotherapy response for future investigations. In conclusion, the 5‐miRNA signature‐based nomogram is useful in predicting radiotherapy response in HNSCC and might become a promising tool to optimize radiation strategies.

## Introduction

Head and neck squamous cell carcinoma (HNSCC), with an overall five‐year survival rate at approaching almost 50 percent, has been considered as one of the most common malignancies in recent years. Several regimens have been put into clinical practice in HNSCC treatment, including surgery, chemotherapy, and radiotherapy (RT).

Radiotherapy is one of the mainstay treatments of HNSCC, and its success rate remains increasing gradually. It was reported that nearly 75 percent patients with HNSCC benefit from radiotherapy, including both neoadjuvant and postoperative adjuvant radiotherapies 1. Nevertheless, a small proportion of patients might be radiation‐resistant, and even suffer from its toxicities, such as disorders on swallowing, salivation, hearing, and cosmetic integrity 2. In some cases, radiotherapy may pose some post‐therapy burdens to these patients and affect their quality of life 3, 4.

However, it remains unsolved to identify patients insensitive to radiotherapy in real‐life clinical practice. Specially, novel methods that can reliably predict individual response to radiotherapy are much warranted.

MicroRNAs (miRNAs), the evolutionarily conservative single‐stranded noncoding RNAs, exert inhibitory effects on protein‐coding genes via targeting the 3′‐untranslated region (UTR) of a target gene, followed by subsequent induction the target degradation of messenger RNAs (mRNA) or suppression of protein translation at the post‐transcriptional level. Recently, several studies focused on the application of miRNAs in the evaluation of radiotherapy response, and a close link has been observed among them in HNSCC. It was reported that an upregulation of miR‐494‐3p was associated with improving response to radiotherapy 5, whereas miR‐196a expression was associated with radio‐resistance and poorer OS in HNSCC 6.

In our study, a 5‐miRNA signature was developed from a large database of The Cancer Genome Atlas (TCGA) to serve as a reliable biomarker for predicting radiotherapy response in HNSCC. Likewise, the biological function of target genes of the 5‐miRNA signature was investigated. In addition, a nomogram which could be put into clinical practice was established.

## Material and Methods

### Data acquisition

Normalized miRNA sequencing data (level 3, reads per kilobase per million mapped reads) as well as medical records of each patient during follow‐up were downloaded/acquired from TCGA database (https://cancergenome.nih.gov). “DESeq,” a package of R software (version 3.3.2, R Foundation for Statistical Computing, Vienna, Austria), was used in the normalization of miRNA‐seq data and to make comparisons between normal mucosa and HNSCC. As a consequence, 56 differential miRNAs with the fold change were two times or more, and significant if FDR (adjust *P* value) < 0.01 was identified. Clinical XML files of each patient were summarized into a .txt document using R package “XML.” Patients with overall survival (OS) <1 month were excluded to avoid the impact of unrelated causes of death.

### Establishment of 5‐miRNA signature with LASSO regression model

The expression level of miRNA was defined as log2 reads per million of total aligned miRNA reads. Based on the overall mortality, X‐tile (version 3.6.1, Yale University School of Medicine, New Haven, CT, USA), an automatic program for calculating cutoffs via Kaplan–Meier survival analysis and log‐rank test 7, was used, and the cutoff of these differential miRNAs was estimated. According to the calculated cutoff, status of each miRNA was assigned as 0 (normalized miRNA expression lower than cutoff) or 1 (normalized miRNA expression higher than cutoff).

In the training set, with the R package “glmnet,” we used the least absolute shrinkage and selection operator method (LASSO, the classical and modified method in Cox regression analysis of high dimensional data^8^) to pick out the most valuable predictable miRNAs and construct a signature which can stratify the patients into high‐risk and low‐risk groups.

### Bioinformatics analysis of target genes

Target genes of the five miRNAs were predicted using variable databases, including miRanda (https://www.microrna.org), PicTar (http://pictar.mdc-berlin.de), mirwalk (http://zmf.umm.uni-heidelberg.de/apps/zmf/mirwalk2), and TargetScan (https://www.targetscan.org), and then, we figured out 738 genes simultaneously predicted in these four databases. After that, we overlapped these genes with genes which were confirmed relative with head and neck cancer and radiotherapy through GeneCards (www.genecards.org). At last, 108 genes were identified. Gene Ontology (GO) analysis as well as KEGG pathway analysis was executed using DAVID Bioinformatics Resources database (https://david.ncifcrf.gov).

### Statistical analysis

Time‐dependent receiver operating characteristic (ROC) curve, a graphical plot that illustrates the diagnostic value of binary classifier system^9^, was performed via R package “pROC.” Youden's index = max (1‐(sensitivity + specificity)). Kaplan–Meier survival analysis was performed to investigate potential risk factors for overall mortality with log‐rank test. A two‐tailed *P*‐value < 0.05 was considered as statistically significant for all analyses executed. Statistical analysis was carried out using SPSS version 16.0 software (SPSS Inc., Chicago, IL, USA).

A nomogram was established in combination with age, T staging, N staging, 5‐miRNA expression, and survival, and Cox regression analysis was performed using SPSS software and R package “rms.” The calibration plot, in which the 45‐degree line was defined as the idealized model, was also performed using “rms.”

## Results

### Differential miRNAs identification and cutoff estimation

miRNA array profiles and corresponding clinical records for patients with HNSCC were obtained from the TCGA database. A total of 553 samples, consisting of 509 carcinomas and 44 normal mucosa specimens, from 509 patients with HNSCC were employed. Among them, 307 patients received external beam radiotherapy after surgical operation, and the radiation dose ranged from 11 Gy to 73 Gy. After normalization, expressions of miRNAs in normal specimens and carcinoma specimens were compared to help overcome the confounding effect of tumor purity, and 56 differential miRNAs (|fold change| ≥2 and FDR < 0.01) were identified (Figure S1). Using X‐tile, the cutoff of each miRNA from these 56 differential miRNAs was calculated in combination with the OS with Kaplan–Meier survival analysis and log‐rank test. As a result, the cutoff expression of miR‐99a, miR‐31, miR‐410, miR‐424, and miR‐495 is 8.5, 5.3, 2.3, 7.5, and 2.3, respectively. The miRNA status was defined as 0 if the miRNA expression level is lower than estimated cutoff and defined as 1 if higher.

### Establishment of a 5‐miRNA signature for predicting clinical outcomes

**Figure 1:**
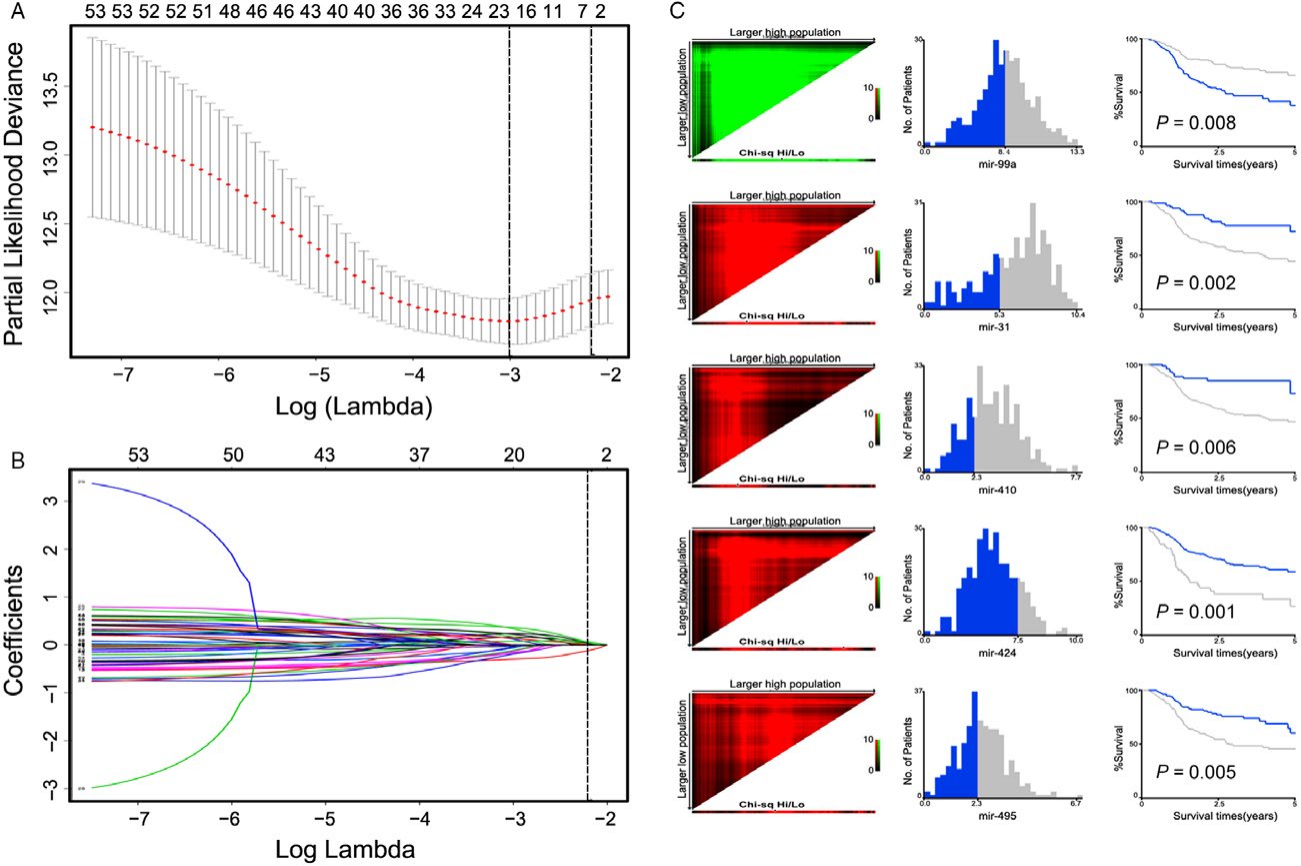
miRNA selection using the least absolute shrinkage and selection operator (LASSO) logistic regression model. (A) Tuning parameter (Lambda, *λ*) selection cross‐validation error curve. The vertical lines were drawn at the optimal values by the minimum criteria and the 1‐SE criteria. We choose the right line by 1‐SE criteria where the value = −1.47 with *λ *= 0.033. (B) The coefficients of 56 differential miRNAs from LASSO model. A vertical line is drawn at the value chosen by 10‐fold cross‐validation. (C) X‐tile analysis of the 5 selected miRNAs. Red indicates inverse association between marker expression and overall survival, whereas green represents direct association.

**Figure 2:**
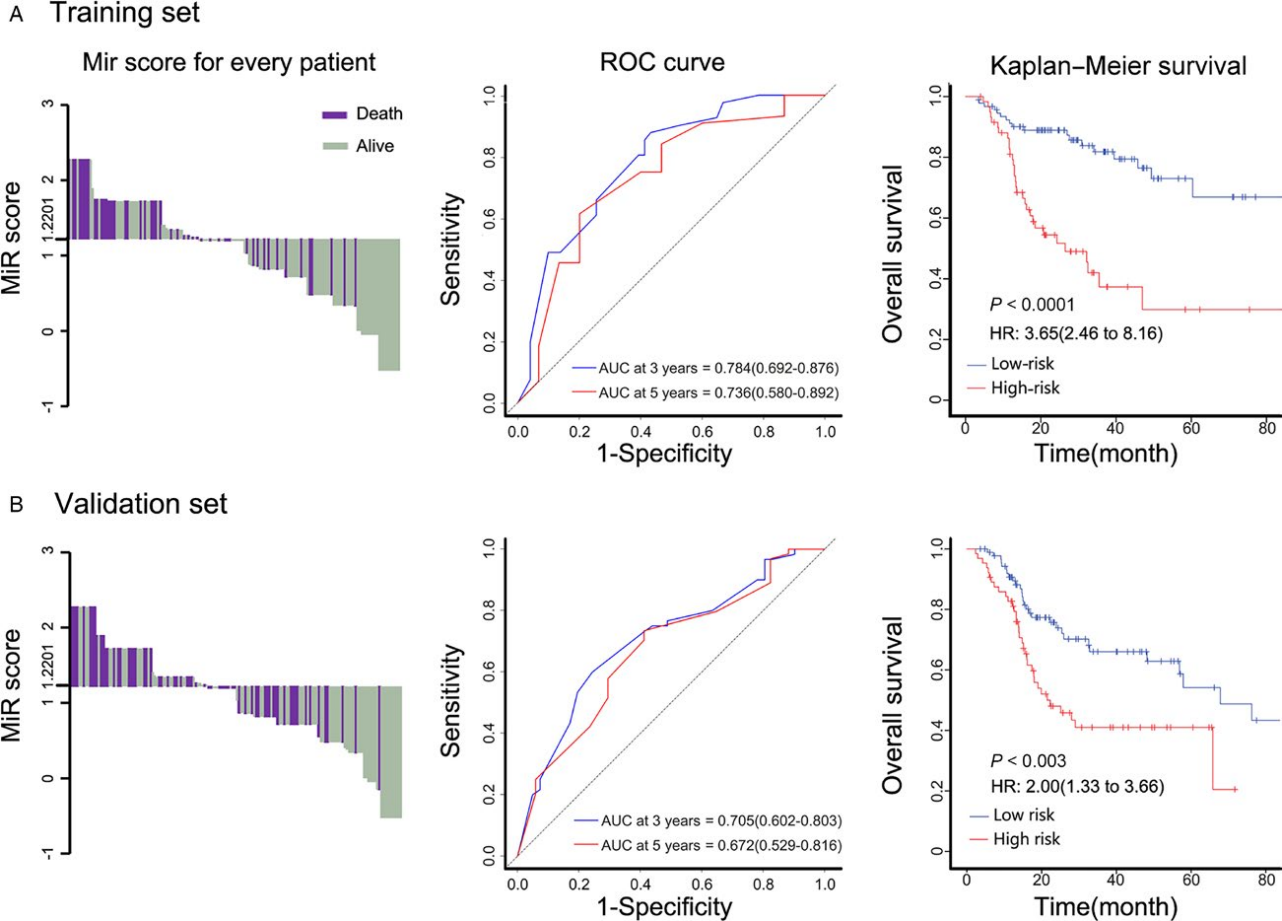
MiR score by the 5‐miRNA signature, time‐dependent ROC curves, and Kaplan–Meier survival in the training and validation sets according to the 5‐miRNA signature. Left panels represent the bar diagrams of every patient's MiR score. It was shown that patients with MiR scores <1.2201 had better survival when compared with those with MiR scores more than 1.2201. Middle panels showed the ROC curves of training set and validation set. Right panels indicate Kaplan–Meier survival analysis of training set and validation set. ROC indicates receiver operating characteristic. AUC indicates area under curve. The AUC was assessed at 3 and 5 years, and the *P* value was acquired through log‐rank test. We calculated *P* values using the log‐rank test.

Three hundred and seven patients receiving radiotherapy were randomly divided into two groups, including a training set (*n* = 154) and a validation set (*n* = 153) (Table 1). In order to predict these patients’ radiotherapy responses, a formula of combined miRNAs was established using LASSO regression model in the training set, where MiR score from 5‐miRNA signature = miR‐99a status × (−0.5289) + miR‐31 status × 0.4797 + miR‐410 status × 0.8658 + miR‐424 status × 0.5513 + miR‐495 status × 0.3789 (Fig. 1A,B), and these five miRNAs linked closely to clinical outcomes of HNSCC RT patients (all *P *<* *0.05; Fig. 1C). A cutoff of 1.2201 was calculated using Youden's index, while favorable sensitivity as well as specificity was shown (62.4% and 75%, respectively). Poorer prognoses were observed in high‐risk patients whose MiR scores were higher than 1.2201, while patients with MiR scores lower than 1.2201 were with favorable prognoses (Fig. 2A, left panel). According to time‐dependent ROC analysis, the prognostic accuracy of the five miRNA signatures is 0.784 at 3 years and 0.736 at 5 years, respectively. It was revealed that the 5‐year survival rate in low‐risk group reached 73%, while significantly lower survival rate (30%) was shown in high‐risk group (hazard ratio [HR] 3.65, 95%CI 2.46–8.16; *P *<* *0.0001; Fig. 2A).

**Table 1:** Clinical covariates for the 307 HNSCC RT patients

		Total	%	Training set	%	Validation set	%	*P*
Age, years, *n* (%)	≤60	152	49.5	109	70.8	94	61.4	0.119
	>60	155	50.5	46	29.9	59	38.6	
Gender, *n* (%)	Male	240	78.2	128	83.1	112	73.2	0.039^a^
	Female	67	21.8	26	16.9	41	26.8	
Clinical T, *n* (%)	1	16	5.2	9	5.8	7	4.6	0.258
	2	71	23.1	38	24.7	33	21.6	
	3	84	27.4	48	31.2	36	23.5	
	4	127	41.4	56	36.4	71	46.4	
Clinical N, *n* (%)	0	112	36.5	54	35.1	58	37.9	0.763
	1	58	18.9	31	20.1	27	17.6	
	2	120	39.1	62	40.3	58	37.9	
	3	5	1.6	3	1.9	2	1.3	
Clinical M, *n* (%)	0	288	93.8	144	93.5	144	94.1	0.247
	1	3	1.0	3	1.9	0	0.0	
Clinical stage, *n* (%)	I	7	2.3	4	2.6	3	2.0	0.396
	II	33	10.7	17	11.0	16	10.5	
	III	55	17.9	33	21.4	22	14.4	
	IV	204	66.4	96	62.3	107	69.9	
Tumor grade, *n* (%)	1	26	8.5	11	7.1	15	9.8	0.518
	2	178	58.0	84	54.5	94	61.4	
	3	79	25.7	41	26.6	38	24.8	
	4	7	2.3	5	3.2	2	1.3	
Survival time, month (mean ± SD)		23.68	16.3	37.05	22.5	20.3	6.9	0.351
Vital status, *n* (%)	Death	190	61.9	87	56.5	103	67.3	0.06
	Alive	117	38.1	67	43.5	50	32.7	

HNSCC, head and neck squamous carcinoma; RT, radiotherapy. *P*‐value was from chi‐square test.

a *P *< 0.05.

Prediction value of this 5‐miRNA signature was further evaluated in the validation set and the total set of all patients who received radiotherapy. The 3‐year and 5‐year prognostic accuracy of these miRNA signatures were 0.705 and 0.672 in the validation set, and 0.743 and 0.699 in the total set, respectively. Similarly, significantly higher 5‐year survival rates were observed in low‐risk groups, in comparison with high‐risk ones, both in the validation set and in the total set (Fig. 2B; Figure S2).

### The prognostic values of the 5‐miRNA signature in HNSCC patients with/without RT

A proportion of patients with HNSCC may be radioresistant, instead of benefiting from radiotherapy, some of them even suffered from its toxicities 2. Identification of these unfavorable patients may help refining radiation therapy strategy.

**Figure 3:**
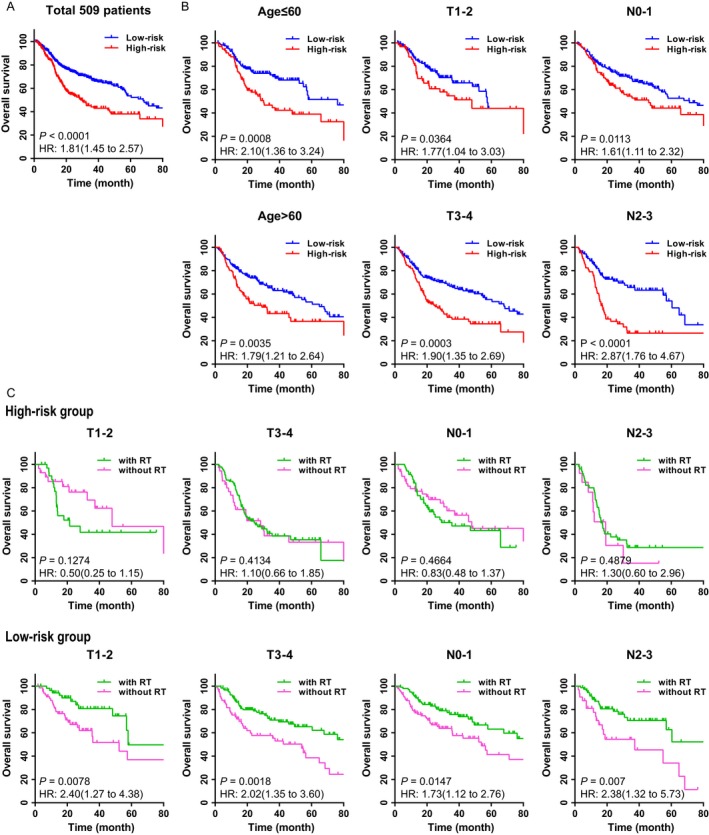
The prognostic values of the 5‐miRNA signature in HNSCC patients with/without RT. (A) Kaplan–Meier analysis of overall survival in 509 patients according to the 5‐miRNA signature. It was observed that the 5‐miRNA signature also had a significant prognostic value in all of 509 HNSCC patients with or without RT. (B) Kaplan–Meier survival analysis in different subgroups of clinical characteristics according to the 5‐miRNA signature. The 5‐miRNA signature was capable of predicting the OS in different clinicopathological factors. (C) Kaplan–Meier survival in 5‐miRNA signature‐based risk group according to patients with/without RT, who were stratified by different clinical characteristics. We calculated *P* values using the log‐rank test. We found in different subgroups, only low‐risk group could get benefit in receiving RT, but high‐risk group had similar survival with or without RT.

We evaluated the prognostic value of the 5‐miRNA signature in a total of 509 patients with HNSCC; patients in low‐risk group predicted by our 5‐miRNA signature generally had better 5‐year OS than those in high‐risk group (Fig. 3A). When further stratified patients according to their clinic‐pathological risk factors, such as age, T stage, N stage, and clinical stage, the 5‐miRNA signature remained as an independent and statistically significant prognostic predictor, except for Stage I‐II patients, which was mainly due to the small proportion. (Figures S3 and S4; Fig. 3B).

To further evaluate the predictive value of the 5‐miRNA signature in HNSCC with radiotherapy, patients were divided into different groups based on the 5‐miRNA signature, and the responses to radiotherapy were tracked. It was revealed that in different subgroups, such as patients with various T stage and N stage (which was often used as the basis to make decision on radiotherapy), the responses to radiotherapy differed in distinguished 5‐miRNA signature‐based groups. In high‐risk group, patients did not benefit from radiotherapy (all *P *>* *0.05; Fig. 3C, upper panels), while better clinical outcomes of radiotherapy were observed in low‐risk patients (all *P *>* *0.05; Fig. 3C, lower panels). These results suggested that high‐risk patients tend to be more insensitive to radiotherapy when compared with low‐risk ones.

As HPV‐positive HNSCC tumors and laryngeal carcinomas are already known to show superior response to standard therapy which typically including radiotherapy and better survival, so we excluded the HPV+ HNSCC tumors (HPV positivity was defined by p16 staining) and laryngeal carcinomas (72 HPV+ and larynx tumors in the low‐risk group and 33 HPV+ and larynx tumors in the high‐risk group) and then repeated our analysis in the rest HNSCC RT patients (*n* = 202). As a consequence, the 5‐miRNA signature remained as an independent prognostic factor for the specific HNSCC RT patients (Figure S5A). Likewise, higher survival rate was observed in the low‐risk group with RT in comparison with those without RT (Figure S5C). Therefore, we can correctly draw the conclusion the low‐risk group produced by our original 5‐miRNA signature did not overrepresent for HPV‐positive tumor and laryngeal carcinoma (Figure S5). Moreover, with the exclusion of HPV‐positive HNSCC and laryngeal carcinomas, our 5‐miRNA signature is also predictive for overall survival by RT treatment.

### Establishment of a nomogram to predict the overall survival in HNSCC with RT

**Figure 4:**
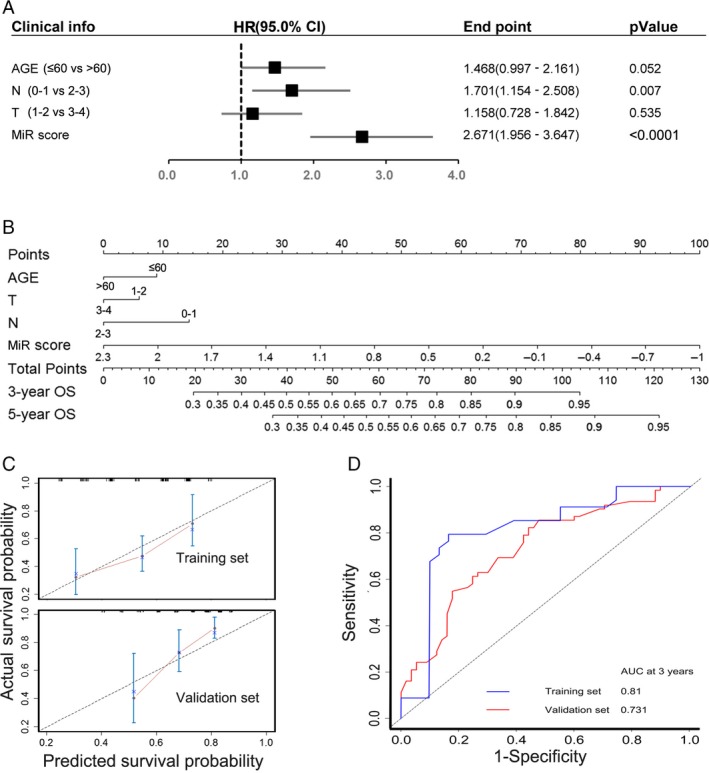
Nomogram to predict the overall survival in HNSCC patients with radiotherapy. (A) Cox multivariate regression with clinical information and MiR score for survival. (B) Nomogram for predicting the 3 and 5 years overall survival in HNSCC with radiotherapy. (C) Calibration plots of the nomogram show predicted 3‐year outcomes are close to the real outcomes in the training and validation sets. The 45‐degree line means the real outcomes. (D) Time‐dependent ROC curves by nomograms for 3‐year overall survival in the training and validation sets.

In order to construct a visual scale to predict the clinical outcomes of patients with HNSCC receiving radiotherapy, multivariate Cox regression analysis was performed to evaluate the relevant variables in predicting overall survival, including the MiR score, age, and TNM staging. It was revealed that except for MiR score, only N stage was correlated with the prognosis of patients with HNSCC receiving radiotherapy (Fig. 4A). Nevertheless, age and T stage were traditionally considered as risk factors for adjuvant radiotherapy in clinical practice 10, 11. As a result, we constructed a nomogram to predict the clinical outcomes of 307 HNSCC patients with radiotherapy, and variables including age, T staging, and N staging were taken into consideration (Fig. 4B).

Nomogram is a simple tool in prognosis evaluation. Firstly, a vertical line could be drawn from each factor to the “Points” line to get a score in the nomogram. Then, each factor's score was added to acquire the total points, which could be used to estimate survival probability. For example, consider a 62‐year‐old T2N1MX HNSCC patient with MiR score equal to 0.8 who received radiotherapy, the total points for this patient is 0 + 9 + 14 + 45 = 65. According to the nomogram, the estimated 3‐year OS is approximately 70–75%, while 5‐year OS is approximately 55–60%. The accuracy of the nomogram is 0.81 and 0.731 at 3 years in the training set and validation set, respectively (Fig. 4D). According to the calibration plot of both sets, the prediction accuracy of the nomogram was comparable to the actual situation (Fig. 4C).

### Target genes prediction and bioinformatics analysis of the 5‐miRNA signature

In order to further investigate the cellular biological functions and potential mechanisms of the 5‐miRNA signature, we predicted target genes of the five miRNAs from four databases, including miRanda, mirwalk, TargetScan, and PicTar. A total of 738 overlapped genes were observed.

To narrow down the range and to ensure that these predicted genes do play roles in patients with HNSCC receiving radiation therapy, we overlapped these 738 predicted genes with 3968 HNSCC relative genes and 4378 radiotherapy‐related genes which were both achieved from GeneCards. At last, we got 118 target genes, which might partially explain the potential mechanisms of the five miRNAs in affecting the prognosis of HNSCC patients with radiotherapy.

**Figure 5:**
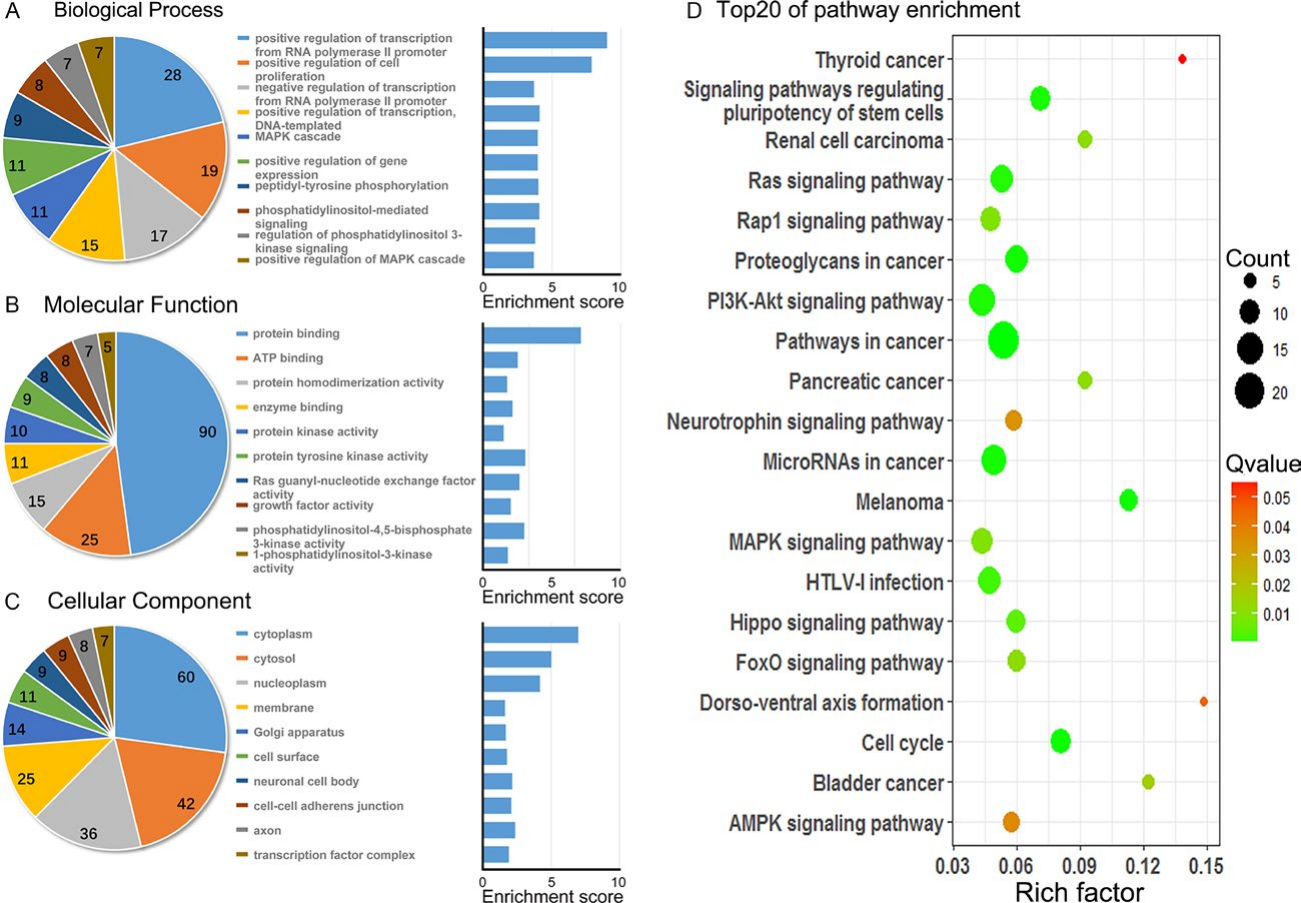
Gene Ontology and pathway analysis of the predicted target gene from five selected miRNAs. (A–C) Top 10 Gene Ontology terms in three domains of the predicted gene. The pie plot means the number of target gene in each term. Enrichment = −log10 (adjust *P* value). (D) Top 20 pathways of the predicted genes. Rich factor = enrichment level. The magnitude of the pots = numbers of gene. The color classification = Qvalue (adjust *P* value).

David Bioinformatics database (https://david.ncifcrf.gov) was used to execute Gene Ontology (GO) analysis and pathway analysis. The results are listed in Table 1. GO analysis consisted of three domains, including biological process (BP), molecular function (MF), and cellular component (CC). It was found that these genes were mainly associated with *gene transcription and cell proliferation* in terms of BP, and involved in MF such as *protein‐binding and protein tyrosine kinase activity* and played a role in constructing *cytoplasm and cytosol* in CC (Fig. 5A–C; Table S1). Pathway analysis indicated that these genes mainly enriched in malignancy‐related pathways (Table S2), including *Pathways in cancer, Cell cycle, Melanoma, Proteoglycans in cancer, microRNAs in cancer,* and so forth (Fig. 5D). In addition, we assumed that genes such as *FGF9, FGF2, FGFR1, FGFR3, HBEGF, IGF1, TGFB2, VEGFA, and MAP2K1* might play crucial roles in HNSCC. However, our target gene prediction findings remain as speculative, awaiting further validation.

## Discussion

Although radiation therapy has been widely used in the treatment of HNSCC, and about 75% patients benefit from it, a subset of patients are still resistant to radiotherapy 2. For the radioresistant subset of patients, radiotherapy has no benefit and may also introduce undesirable side effects and compromises their quality of life.

A number of researchers were trying to figure out methods in predicting radiotherapy effects in HNSCC. In a retrospective study of Aaron et al. 12, which focused on the relationship between HNSCC body composition and radiotherapy, pre‐RT body mass index (BMI) played roles in predicting RT response. Likewise, HPV‐positive tumors are more radiosensitive, when compared with HPV‐negative ones 13. Recent study 14 showed the expression of 13 genes was closely correlated with the postradiotherapy prognosis of HPV‐negative HNSCC. However, these studies only illustrated the possible factors relevant to radiotherapy effects, and predictive biomarkers for clinical practice were still lacking.

In recent years, the relation between miRNAs and radiotherapy responses aroused much concern in cancer. For example, Liu et al. 15 figured out a 5‐miRNA radiosensitive signature through the comparison between radiosensitive and radioresistant cell lines. However, it was not equal to the real condition in tumor microenvironment in vivo. Furthermore, comparisons were only made between patients radiated with a complete response and with tumor progression on the expression of each miRNA in this signature through TCGA. Nevertheless, we found only one (mir‐150) of the above previously identified miRNAs (miR‐16, miR‐29b, miR‐150, miR‐1254, and Let‐7e) was related to the clinical outcomes of HNSCC RT (Figure S6), while another five miRNAs (miR‐99a, miR‐31, miR‐410, miR‐424, and miR‐495) in our study significantly correlated with HNSCC radiotherapy effects response. All these five miRNAs played roles in the predicting the progression of HNSCC radiotherapy. Among them, a positive correlation was observed between the expression and prognosis of miR‐99a, while negative links were found in the remaining miRNAs. All these five miRNAs could be used to divide patients with HNSCC receiving RT into two groups with distinct clinical outcomes (Fig. 1C).

Among these miRNAs, miR‐99a regulates DNA damage in prostate cancer cells, thus influence individual sensitivity to radiation 16. Increasing level of miR‐31 strongly correlated with low survival rate in Chinese patients with lung squamous cell carcinoma, via targeting the tumor suppressor *DICER1* 17. MiR‐410 acts as oncogene in non‐small‐cell lung carcinoma patients through suppressing *SLC34A2*, thus activating Wnt/*β*‐catenin pathway. Furthermore, miR‐424 was observed to impair ubiquitination to activate *STAT3* and then promote the progression of prostate tumor 18. MiR‐495 facilitates the progression of breast cancer via repressing *JAM‐A* 19.

However, studies are still lacking for the investigation of correlation between those miRNAs expression and HNSCC radiotherapy. To the best of our knowledge, this is the first study to investigate the potential roles of these miRNAs in the prediction of radiotherapy sensitivity in patients with HNSCC. In addition, target genes of these miRNAs were predicted via several databases; both GO analysis and KEGG analysis were executed. As a result, most of these target genes played roles in crucial cell function and pathways, including “*protein‐binding, Pathways in cancer, Cell cycle, Melanoma, Proteoglycans in cancer, microRNAs in cancer,* and so on.” Target genes involved in these top enriched pathways included fibrosis‐related genes (such as *FGF9, FGF2, FGFR1, and FGFR3*), immune‐related genes (such as *TGFB2*), tumor‐relevant genes (such as *MAP2K1*), and so forth. Our study might provide novel therapeutic targets in patients with HNSCC, and the potential mechanisms of these miRNAs in HNSCC radiotherapy sensitivity warrant further study.

Our study revealed that in order to reduce patients’ suffering and medical costs, the prescription of radiotherapy might differ between low‐risk patients and high‐risk ones. Early miRNA detection is recommended before the initiation of radiation therapy, and tailored radiotherapy strategy or alternative therapy is indicated in patient with high‐risk miRNAs status.

Comparisons were drawn between TNM staging and model analysis. In our population, T stage did not do well in predicting the survival of HNSCC radiotherapy, which means the TNM staging might not be precise enough in assessing the response of radiotherapy in HNSCC. Nevertheless, our 5‐miRNA signature is capable of predicting clinical outcomes, when compared with other clinical variables such as age and TNM staging (Fig. 4A). Differed from other studies, a nomogram was established for clinical practice simultaneously, which might be used in real‐life clinical practice and promote the recognition of radiotherapy‐responsive status. This nomogram might be used not only to assist physicians in the decision‐making process but also to act as a prognostic factor for patients with HNSCC.

There also existed some limitations. First of all, further clinical trials are needed to validate the predictive accuracy of our nomogram in HNSCC patients with radiotherapy. In addition, further studies to investigate the potential mechanisms of these miRNAs both in vitro and in vivo were lacked, as well as prospective studies.

In conclusion, the 5‐miRNA signature may serve as a novel and reliable biomarker for prediction of clinical outcomes in patients with HNSCC receiving radiotherapy. Likewise, the nomogram based on 5‐miRNA signature might become a promising tool in the decision‐making on radiotherapy and prognosis estimation.

## Conflict of Interest

None declared.

## Supporting information


**Figure S1.** HEATMAP 0F 56 miRNA.
**Figure S2.** Mir score by the 5‐miRNA signature, time‐dependent ROC curves and Kaplan Meier survival in total adiotherapy sets.
**Figure S3.** The 5‐miRNA signature was independent. No significant difference between different groups in every clinical characteristic.
**Figure S4.** The prognostic values of the 5‐miRNA signature for HNSCC patients with/without RT in different clinical stage.
**Figure S5.** The prognostic values of the 5‐miRNA signature in HNSCC patients(excluding HPV+ HNSCC and laryngeal carcinomas) with/without RT.
**Figure S6.** X‐tile plots of other 5 miRNA from Liu’s study.
**Table S1.** Top 10 Gene oncology terms in 3 domains of the predicted genes.
**Table S2.** Pathway analysis of predicted genes.

## References

[1] Gregoire, V. , J. A. Langendijk , and S. Nuyts . 2015 Advances in radiotherapy for head and neck cancer. J. Clin. Oncol. 33:3277–3284. 26351354 10.1200/JCO.2015.61.2994

[2] Wang, X. , C. Hu , and A. Eisbruch . 2011 Organ‐sparing radiation therapy for head and neck cancer. Nat. Rev. Clin. Oncol. 8:639–648. 21788974 10.1038/nrclinonc.2011.106

[3] Caudell, J. J. , J. F. Torres‐Roca , R. J. Gillies , H. Enderling , S. Kim , A. Rishi , et al. 2017 The future of personalised radiotherapy for head and neck cancer. Lancet Oncol. 18:e266–e273. 28456586 10.1016/S1470-2045(17)30252-8 PMC7771279

[4] Lacas, B. , J. Bourhis , J. Overgaard , Q. Zhang , V. Grégoire , M. Nankivell , et al. 2017 Role of radiotherapy fractionation in head and neck cancers (MARCH): an updated meta‐analysis. Lancet Oncol. 18:1221–1237. 28757375 10.1016/S1470-2045(17)30458-8 PMC5737765

[5] Weng JH, Y. C. , L. YC , L. CW , C. WW , and K. YL . 2016 miR‐494‐3p induces cellular senescence and enhances radiosensitivity in human oral squamous carcinoma cells. Int. J. Mol. Sci. 17:1092. 10.3390/ijms17071092 PMC4964468 27399693

[6] Suh, Y. E. , N. Raulf , J. Gaken , K. Lawler , T. G. Urbano , J. Bullenkamp , et al. 2015 MicroRNA‐196a promotes an oncogenic effect in head and neck cancer cells by suppressing annexin A1 and enhancing radioresistance. Int. J. Cancer 137:1021–1034. 25523631 10.1002/ijc.29397

[7] Camp, R. L. , M. Dolled‐Filhart , and D. L. Rimm . 2004 X‐tile: a new bio‐informatics tool for biomarker assessment and outcome‐based cut‐point optimization. Clin. Cancer Res. 10:7252–7259. 15534099 10.1158/1078-0432.CCR-04-0713

[8] Tibshirani, R. 1997 The lasso method for variable selection in the Cox model. Stat. Med. 16:385–395. 9044528 10.1002/(sici)1097-0258(19970228)16:4<385::aid-sim380>3.0.co;2-3

[9] Heagerty, P. J. , T. Lumley , and M. S. Pepe . 2000 Time‐dependent ROC curves for censored survival data and a diagnostic marker. Biometrics 56:337–344. 10877287 10.1111/j.0006-341x.2000.00337.x

[10] Rocha, H. , L. Khouri , M. C. Lopes , J. Dias , and B. Ferreira . 2016 Treatment failure prediction for head‐and‐neck cancer radiation therapy. Cancer Radiother. 20:268–274. 27321413 10.1016/j.canrad.2016.02.012

[11] Bobdey, S. , G. Balasubramaniam , and P. Mishra . 2016 Nomogram prediction for survival of patients with oral cavity squamous cell carcinoma. Head Neck 38:1826–1831. 27227458 10.1002/hed.24507

[12] Grossberg, A. J. , S. Chamchod , C. D. Fuller , A. S. Mohamed , J. Heukelom , H. Eichelberger , et al. 2016 Association of body composition with survival and locoregional control of radiotherapy‐treated head and neck squamous cell carcinoma. JAMA Oncol. 2:782–789. 26891703 10.1001/jamaoncol.2015.6339 PMC5080910

[13] Mirghani, H. , F. Amen , Y. Tao , E. Deutsch , and A. Levy . 2015 Increased radiosensitivity of HPV‐positive head and neck cancers: molecular basis and therapeutic perspectives. Cancer Treat. Rev. 41:844–852. 26476574 10.1016/j.ctrv.2015.10.001

[14] Foy, J. P. , L. Bazire , S. Ortiz‐Cuaran , S. Deneuve , J. Kielbassa , E. Thomas , et al. 2017 A 13‐gene expression‐based radioresistance score highlights the heterogeneity in the response to radiation therapy across HPV‐negative HNSCC molecular subtypes. BMC Med. 15:165. 28859688 10.1186/s12916-017-0929-y PMC5580222

[15] Liu, N. , R. J. Boohaker , C. Jiang , J. R. Boohaker , and B. Xu . 2015 A radiosensitivity MiRNA signature validated by the TCGA database for head and neck squamous cell carcinomas. Oncotarget 6:34649–34657. 26452218 10.18632/oncotarget.5299 PMC4741479

[16] Mueller, A. C. , D. Sun , and A. Dutta . 2013 The miR‐99 family regulates the DNA damage response through its target SNF2H. Oncogene 32:1164–1172. 22525276 10.1038/onc.2012.131 PMC3407337

[17] Tan, X. , W. Qin , L. Zhang , J. Hang , B. Li , C. Zhang , et al. 2011 A 5‐microRNA signature for lung squamous cell carcinoma diagnosis and hsa‐miR‐31 for prognosis. Clin. Cancer Res. 17:6802–6811. 21890451 10.1158/1078-0432.CCR-11-0419

[18] Ke, X. , Y. Yuan , C. Guo , Y. Yang , Q. Pu , X. Hu , et al. 2017 MiR‐410 induces stemness by inhibiting Gsk3beta but upregulating beta‐catenin in non‐small cells lung cancer. Oncotarget 8:11356–11371. 28076327 10.18632/oncotarget.14529 PMC5355270

[19] Cao, M. , W. Nie , J. Li , Y. Zhang , X. Yan , X. Guan , et al. 2014 MicroRNA‐495 induces breast cancer cell migration by targeting JAM‐A. Protein Cell. 5:862–872. 25070379 10.1007/s13238-014-0088-2 PMC4225486

