# Supplementary material for: Prediction of radiotherapy response with a 5‐microRNA signature‐based nomogram in head and neck squamous cell carcinoma
Source: Cancer Med. 2018 Feb 23;7(3):726–35. doi: 10.1002/cam4.1369 (PMC5852342; doi:10.1002/cam4.1369)
Supplement: Supplementary file 1 — Figure S1. HEATMAP 0F 56 miRNA. Figure S2. Mir score by the 5‐miRNA signature, time‐dependent ROC curves and Kaplan Meier survival in total adiotherapy sets. Figure S3. The 5‐miRNA signature was independent. No significant difference between different groups in every clinical characteristic. Figure S4. The prognostic values of the 5‐miRNA signature for HNSCC patients with/without RT in different clinical stage. Figure S5. The prognostic values of the 5‐miRNA signature in HNSCC patients(excluding HPV+ HNSCC and laryngeal carcinomas) with/without RT. Figure S6. X‐tile plots of other 5 miRNA from Liu’s study. Table S1. Top 10 Gene oncology terms in 3 domains of the predicted genes. Table S2. Pathway analysis of predicted genes. [file CAM4-7-726-s001.docx]

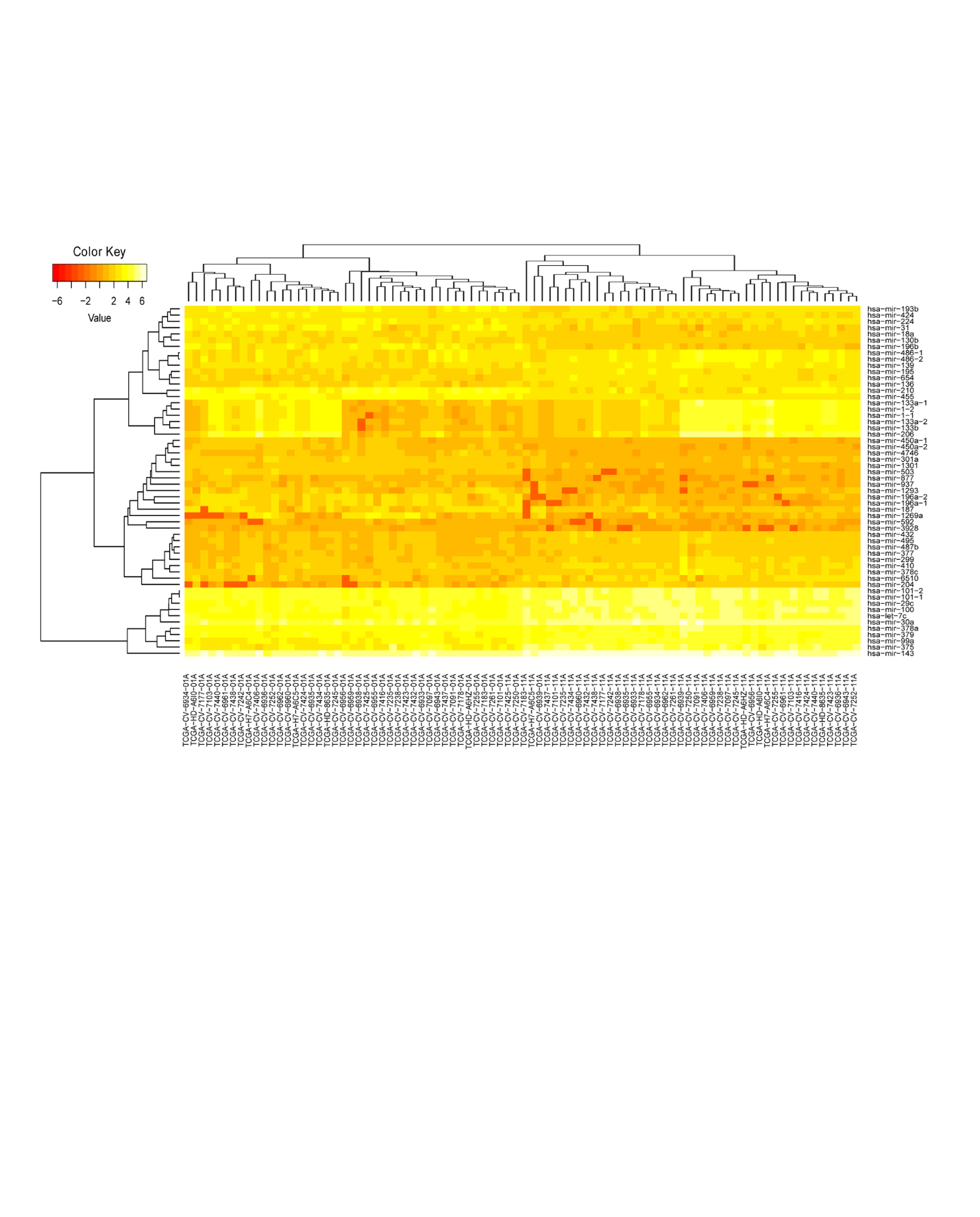


**Figure S1. HEATMAP 0F 56 miRNA**

Hierarchical clustering of tumor tissues and adjacent normal mucosa with the 56 differentially expressed miRNAs. Every row represents an individual miRNA, and each column represents an individual sample. The dark orange color indicates low expression levels whereas white indicates high expression levels.

**
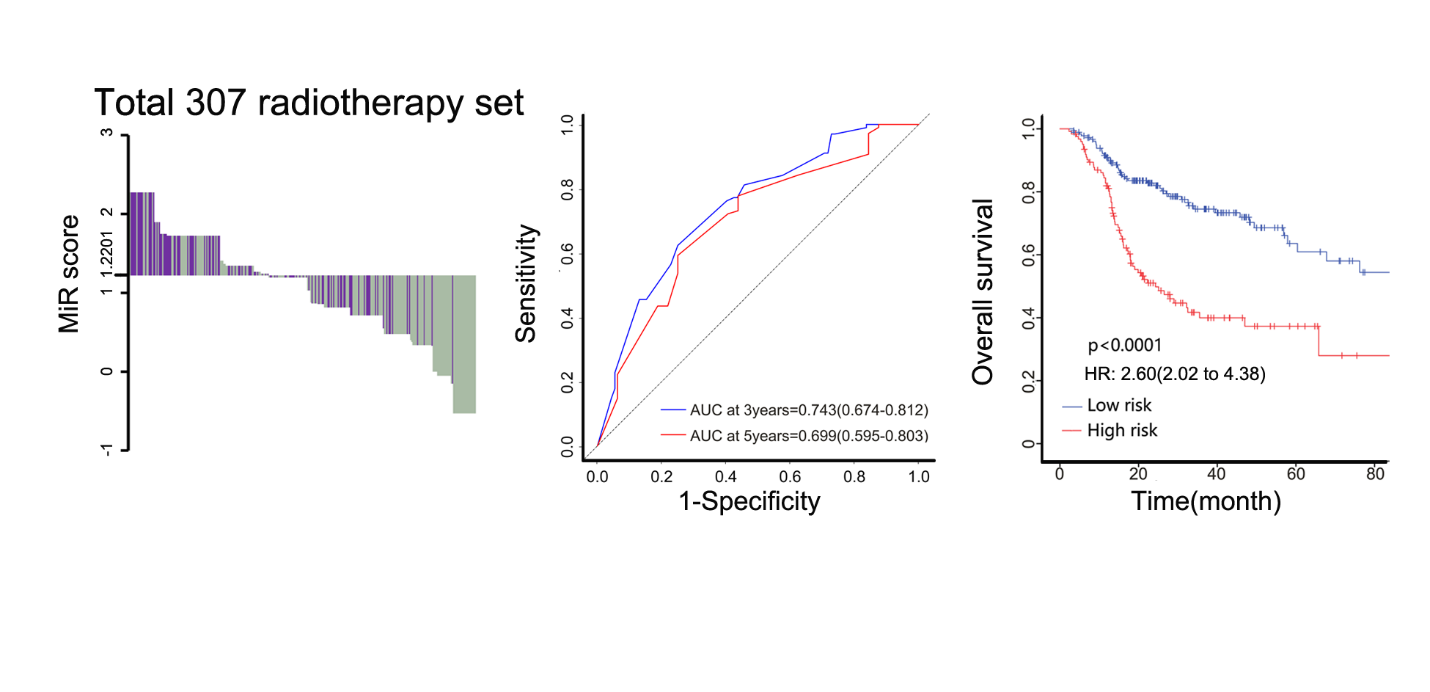
**

**Figure S2. Mir score by the 5-miRNA signature, time-dependent ROC curves and Kaplan Meier survival in total radiotherapy sets.**

Left panels represent the bar diagrams of every patient’s MiR score. It was shown that patients with MiR scores less than 1.2201 had better survival, when compared with those with MiR scores more than 1.2201. Middle panels showed the ROC curves of total 307 patients on radiotherapy. Right panels indicates Kaplan-Meier survival analysis of total 307 patients on radiotherapy.


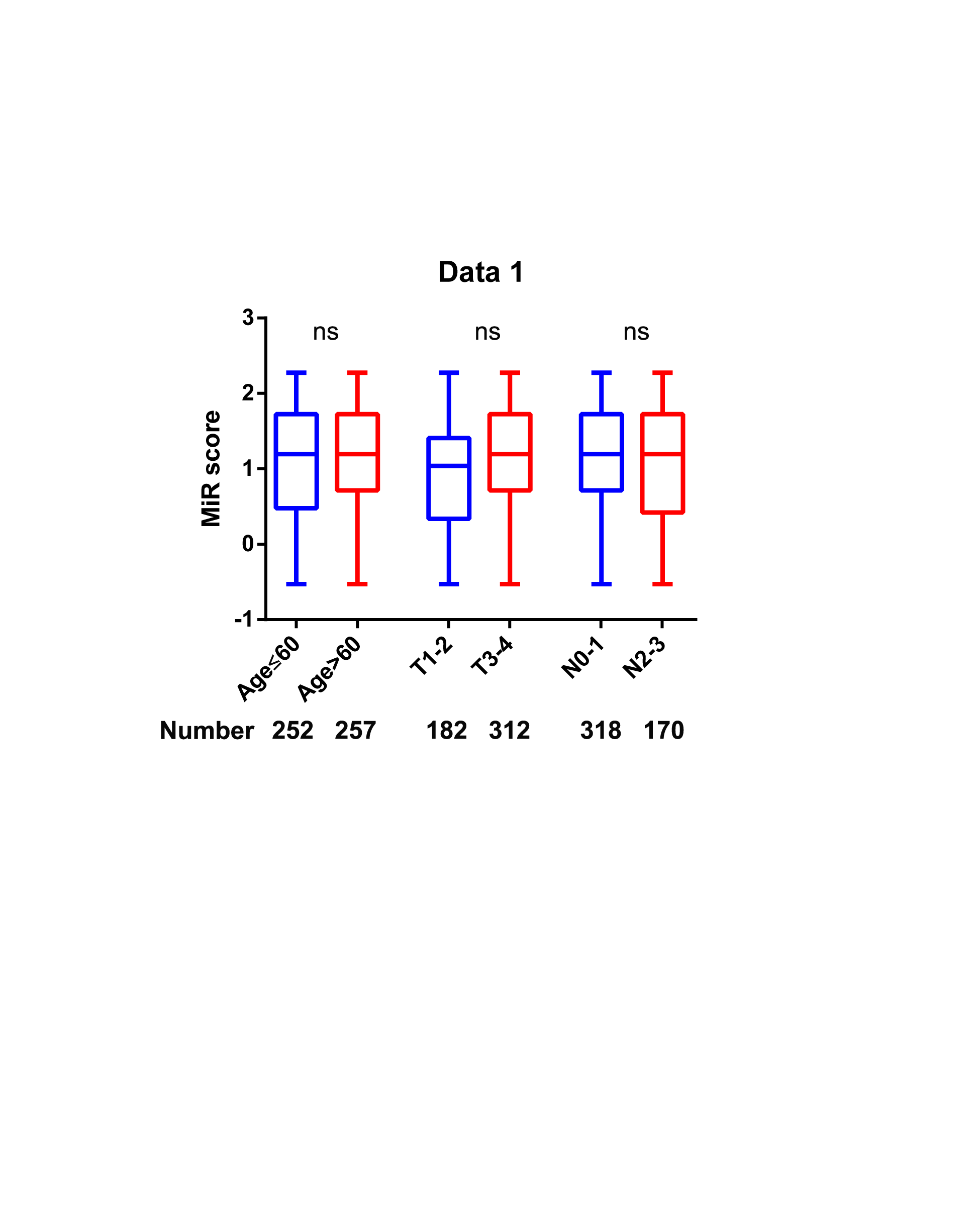


**Figure S3. The 5-miRNA signature was independent. No significant difference between different groups in every clinical characteristic.** Number means the number of samples (n) for each of the categories presented.


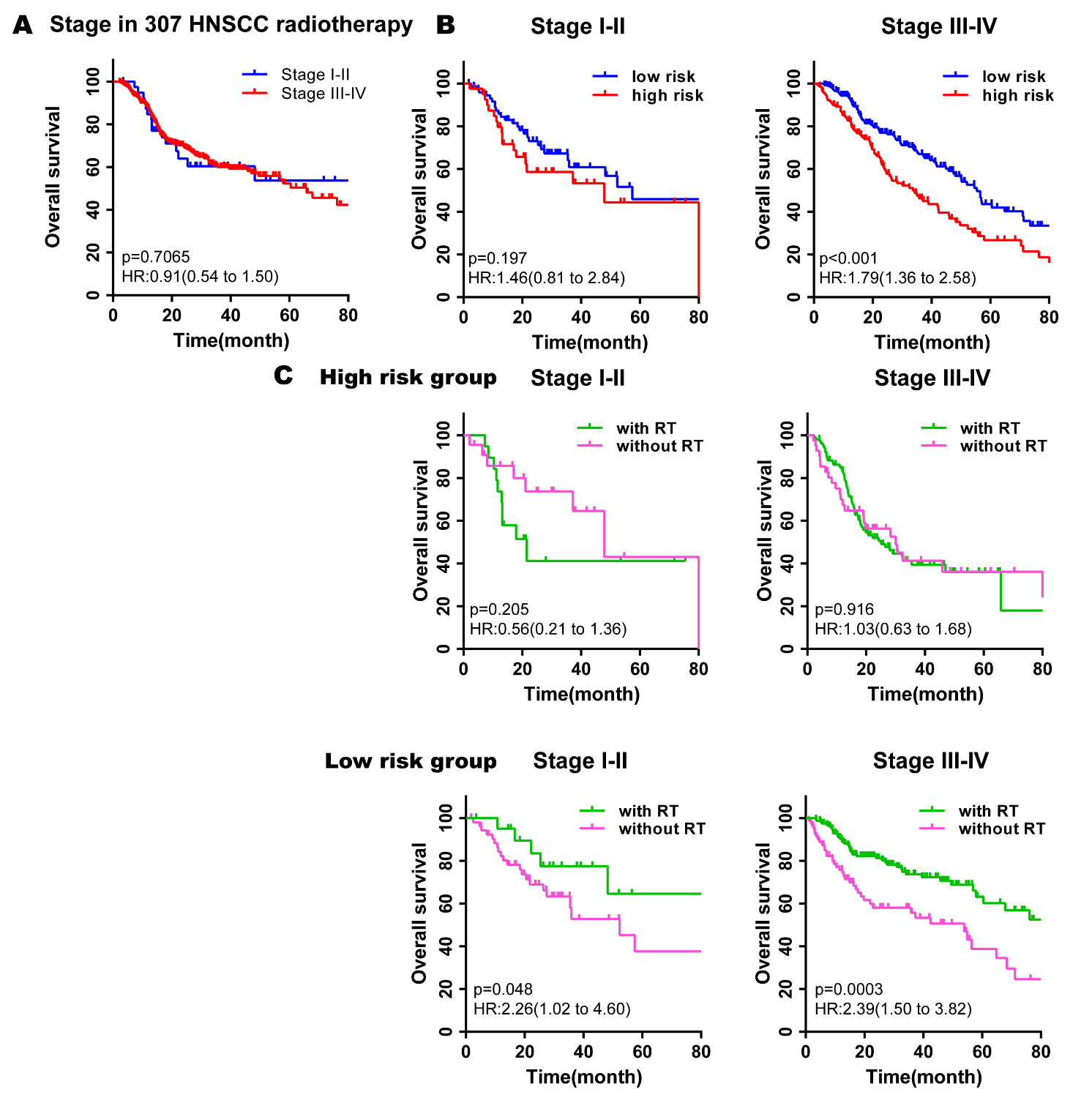


**Figure S4. The prognostic values of the 5-miRNA signature for HNSCC patients with/without RT in different clinical stage.**

A. There were no difference about overall survival between Stage 1-2 group and Stage 3-4 group in HNSCC with radiotherapy. B. The 5-miRNA signature only had predictive value in in stage III-IV patients, but not in stage I-II patients. C. Low risk group got benefit in receiving RT in Stage I-II and III-IV group, but high-risk group had similar survival with or without RT.


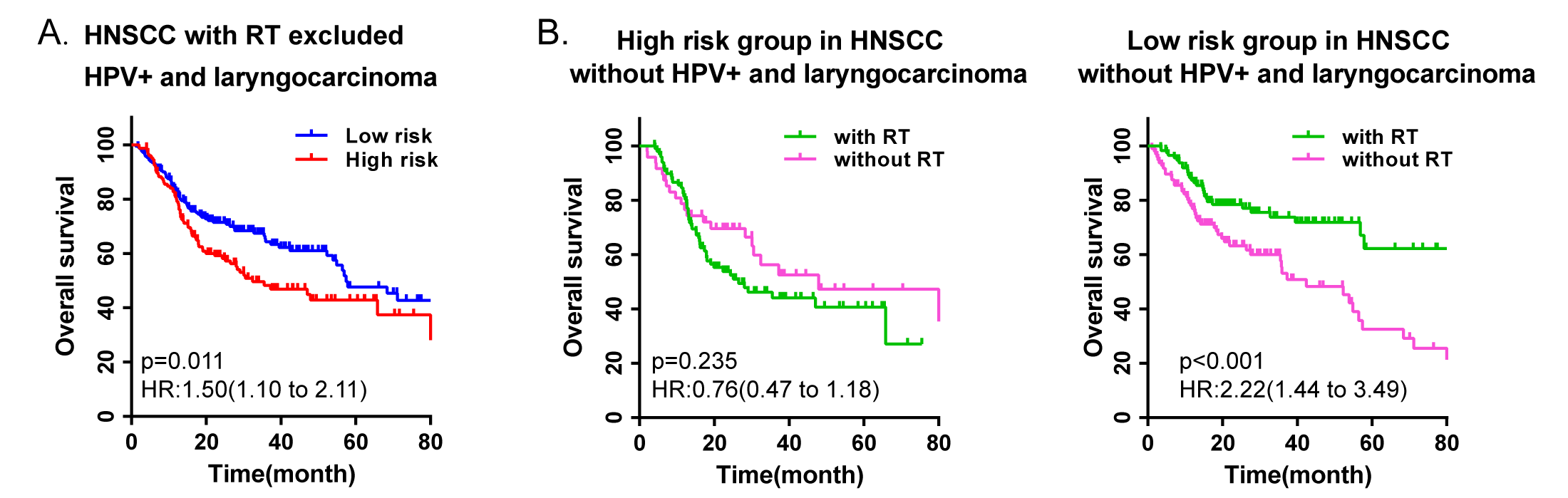


**Figure S5. The prognostic values of the 5-miRNA signature in HNSCC patients(excluding HPV+ HNSCC and**  **laryngeal carcinomas) with/without RT.**

A. Kaplan-Meier analysis of overall survival in all patients without HPV+ HNSCC and laryngeal carcinomas according to the 5-miRNA signature. It was observed that the 5-miRNA signature had a significant prognostic value in HNSCC RT patients excluding HPV+ HNSCC and laryngeal carcinomas. B. Kaplan-Meier survival in 5-miRNA signature based risk group according to patients (excluding HPV+ HNSCC and laryngeal carcinomas) with/without RT. We calculated *p* values using the log-rank test. We found only low-risk group could get benefit in receiving RT, but high-risk group had similar survival with or without RT.


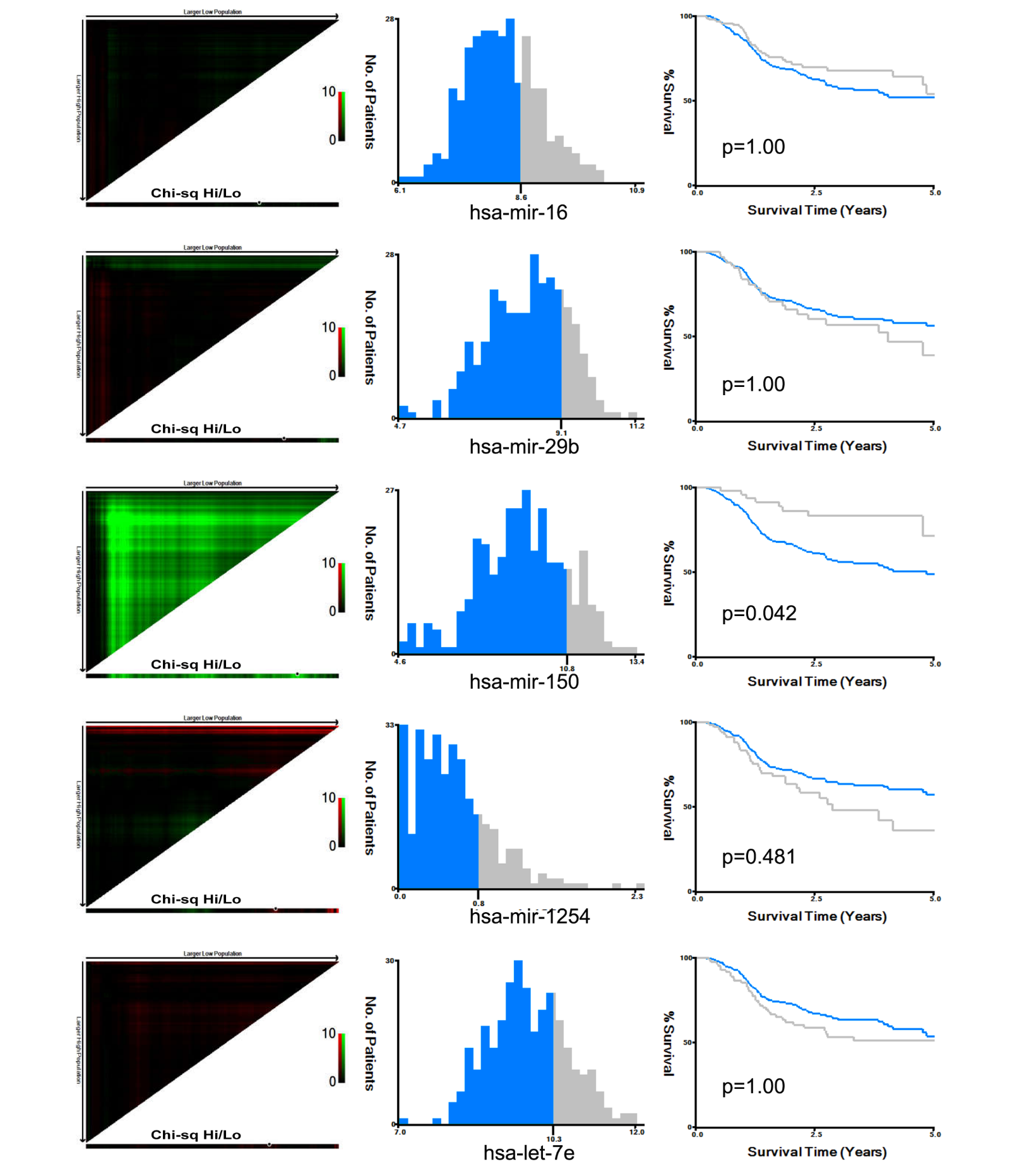


**Figure S4. X-tile plots of other 5 miRNA from Liu’s study .**

| **Supplemental table s1. Top 10 Gene oncology terms in 3 domains of the predicted genes** | | | |
| --- | --- | --- | --- |
| **Domain** | **Term** | **adjust p value** | **Gene** |
| Biological Process | GO:0045944~positive regulation of transcription from RNA polymerase II promoter | 1.02E-09 | THRB, BMPR2, CASK, EGLN1, NR2C2, CDKN2B, PTH, ETV1, YAP1, RARB, TCF4, MYB, FGF2, CD28, PLAG1, SMAD7, IGF1, DLL1, STAT1, ATF6, CDH13, EYA1, ETS1, ETS2, VEGFA, LRP6, NHLH2, RBPJ |
|  | GO:0008284~positive regulation of cell proliferation | 1.36E-08 | FGFR1, FGFR3, FGF7, FGF9, IGF1, DLL1, PURA, TGFB2, EDNRB, CRKL, ETS1, CCND2, VEGFA, HBEGF, PRKAA1, YAP1, RARB, FGF2, ALOX12 |
|  | GO:0045893~positive regulation of transcription, DNA-templated | 8.04E-05 | RET, FGF7, MAP2K1, IGF1, TBP, STAT1, FZD4, ETS1, ETS2, SMARCA5, LRP6, MYB, TCF4, FGF2, ZFHX3 |
|  | GO:0048015~phosphatidylinositol-mediated signaling | 8.41E-05 | FGFR1, FGFR3, FGF7, FGF9, IGF1, HBEGF, FGF2, CD28 |
|  | GO:0018108~peptidyl-tyrosine phosphorylation | 9.95E-05 | FGFR1, RET, FGFR3, FGF7, MAP2K1, FGF9, HBEGF, FGF2, WEE1 |
|  | GO:0000165~MAPK cascade | 1.11E-04 | FGFR1, RET, FGFR3, FGF7, MAP2K1, FGF9, CAMK2G, NF1, HBEGF, SPRED1, FGF2 |
|  | GO:0010628~positive regulation of gene expression | 1.11E-04 | PLAG1, ACTC1, MAP2K1, FGF9, SLC6A4, CYP26B1, VEGFA, PRKAA1, TGFB2, CD28, ALOX12 |
|  | GO:0014066~regulation of phosphatidylinositol 3-kinase signaling | 1.70E-04 | FGFR1, FGFR3, FGF7, FGF9, HBEGF, FGF2, CD28 |
|  | GO:0000122~negative regulation of transcription from RNA polymerase II promoter | 2.07E-04 | FGFR1, THRB, SMAD7, FGF9, DICER1, CBX2, STAT1, EDNRB, CCND1, ETS2, PTH, VEGFA, RARB, RBPJ, MYB, TCF4, ZFHX3 |
|  | GO:0043410~positive regulation of MAPK cascade | 2.12E-04 | FGFR1, TNFRSF1B, FGFR3, FGF9, IGF1, CDH2, PRKCE |
| Molecular Function | GO:0005515~protein binding | 2.03E-11 | FGF7, THRB, VAPB, DICER1, SLC6A4, CASK, SHOC2, TBP, CBX2, PRKG1, CD2AP, TGFB2, EDNRB, SH2D1A, CDKN2B, SPRED1, YAP1, MYB, FGF2, AKT3, PPP2R1B, CDC6, RET, DLL1, PCM1, PRKCE, WEE1, RAD50, PURA, MFN2, MIB1, CCDC6, CCND1, EYA1, KIF1B, CRKL, CCND2, IGF2R, VEGFA, SMARCA5, KPNA2, ALOX12, FGFR1, FGFR3, LITAF, GLUD1, CAMK2G, CTNND2, BMPR2, BCL2L2, CHEK1, EGLN1, CDH2, NR2C2, IARS, TNFRSF1B, FBXW7, BUB1, FASN, ETV1, PRKAA1, PAFAH1B1, HSPA5, TCF4, ARHGDIA, CD28, PTPRD, MAP2K1, SMAD7, NF1, IGF1, ELAVL1, FZD3, STAT1, FZD4, YWHAE, CDC25A, ATF6, ATP7A, NOTCH2, ATXN2, ETS1, ETS2, CD274, SMPD1, LRP6, NHLH2, WIF1, RBPJ, ZFHX3 |
|  | GO:0004713~protein tyrosine kinase activity | 2.40E-05 | FGFR1, RET, FGFR3, FGF7, MAP2K1, FGF9, HBEGF, FGF2, WEE1 |
|  | GO:0046934~phosphatidylinositol-4,5-bisphosphate 3-kinase activity | 3.16E-05 | FGFR1, FGFR3, FGF7, FGF9, HBEGF, FGF2, CD28 |
|  | GO:0005088~Ras guanyl-nucleotide exchange factor activity | 1.01E-04 | FGFR1, RET, FGFR3, FGF7, FGF9, CAMK2G, HBEGF, FGF2 |
|  | GO:0005524~ATP binding | 1.62E-04 | FGFR1, FGFR3, CAMK2G, GLUD1, DICER1, BMPR2, CASK, CHEK1, PRKG1, IARS, BUB1, PRKAA1, HSPA5, AKT3, CDC6, ACTC1, RET, MAP2K1, PRKCE, RAD50, WEE1, ATP7A, KIF1B, SMARCA5, CLCN5 |
|  | GO:0019899~enzyme binding | 5.82E-04 | CCND1, THRB, VAPB, IGF2R, TBP, EGLN1, HSPA5, PRKCE, STAT1, YWHAE, ZFHX3 |
|  | GO:0008083~growth factor activity | 9.26E-04 | BDNF, FGF7, FGF9, VEGFA, IGF1, HBEGF, FGF2, TGFB2 |
|  | GO:0016303~1-phosphatidylinositol-3-kinase activity | 0.0020 | FGFR1, FGFR3, FGF7, FGF9, FGF2 |
|  | GO:0042803~protein homodimerization activity | 0.0023 | FGFR1, VAPB, CAMK2G, SLC6A4, ELAVL1, BCL2L2, STAT1, FZD4, TGFB2, CDH13, VEGFA, FASN, LRP6, PAFAH1B1, TCF4 |
|  | GO:0004672~protein kinase activity | 0.0055 | CCND1, RET, MAP2K1, BUB1, CASK, PRKAA1, CHEK1, PRKCE, AKT3, WEE1 |
| Cellular Component | GO:0005737~cytoplasm | 1.01E-07 | VAPB, FGF9, DICER1, SHOC2, CASK, TBP, PRKG1, CD2AP, SH2D1A, BDNF, CDKN2B, SPRED1, YAP1, RARB, FGF2, AKT3, CDC6, RET, PCM1, PRKCE, WEE1, PURA, MIB1, CCDC6, CCND1, EYA1, IGF2R, VEGFA, SERPINB2, KPNA2, ALOX12, LITAF, GLUD1, CTNND2, BMPR2, LRIG2, EGLN1, CDH2, IARS, FBXW7, CYP26B1, FASN, BUB1, PRKAA1, ARHGDIA, ACTC1, MAP2K1, SMAD7, NF1, ELAVL1, FZD3, STAT1, CDC25A, ATXN2, CDH13, ETS1, ETS2, RBPJ, PSAT1, ZFHX3 |
|  | GO:0005829~cytosol | 9.34E-06 | FGFR1, CAMK2G, SLC6A4, DICER1, CASK, BCL2L2, EGLN1, CHEK1, PRKG1, IARS, SH2D1A, FBXW7, CDKN2B, FASN, BUB1, RHOBTB1, PRKAA1, PAFAH1B1, SPRED1, YAP1, ARHGDIA, CD28, CDC6, ACTC1, MAP2K1, SMAD7, NF1, ELAVL1, PRKCE, PCM1, STAT1, YWHAE, CDC25A, ATP7A, MFN2, MIB1, CCND1, CRKL, CCND2, PSAT1, KPNA2, ALOX12 |
|  | GO:0005654~nucleoplasm | 6.31E-05 | LITAF, THRB, CAMK2G, SHOC2, TBP, CBX2, CHEK1, NR2C2, IARS, FBXW7, CDKN2B, BUB1, PRKAA1, SPRED1, YAP1, RARB, AKT3, CDC6, SMAD7, ELAVL1, STAT1, RAD50, WEE1, CDC25A, ATF6, NOTCH2, ATXN2, CCND1, EYA1, CCND2, ETS1, ETS2, SMARCA5, RBPJ, ZFHX3, KPNA2 |
|  | GO:0030424~axon | 0.0042 | RET, SCN2A, DICER1, NF1, FZD3, STAT1, YWHAE, TGFB2 |
|  | GO:0043025~neuronal cell body | 0.0067 | ATP7A, TNFRSF1B, RET, BMPR2, LRP6, PAFAH1B1, FZD3, TGFB2, PURA |
|  | GO:0005913~cell-cell adherens junction | 0.0079 | CRKL, SMAD7, VAPB, FASN, HSPA5, CDH2, STAT1, CD2AP, YWHAE |
|  | GO:0005667~transcription factor complex | 0.0121 | SMAD7, ETS1, NHLH2, YAP1, RBPJ, TCF4, ZFHX3 |
|  | GO:0009986~cell surface | 0.0165 | NOTCH2, FGFR3, IGF2R, VEGFA, BMPR2, LRP6, HBEGF, FZD3, HSPA5, FZD4, CD28 |
|  | GO:0005794~Golgi apparatus | 0.0202 | CDC6, FGFR3, FGF7, MAP2K1, LITAF, VAPB, PRKG1, PRKCE, ATP7A, ATF6, ATXN2, FASN, LRP6, AKT3 |
|  | GO:0016020~membrane | 0.0227 | CAMK2G, CDH2, IARS, FASN, BUB1, YAP1, HSPA5, ACTC1, RET, NF1, ELAVL1, PCM1, YWHAE, RAD50, PNPLA6, ATP7A, ATF6, ATXN2, NOTCH2, CCND1, IGF2R, VEGFA, KPNA2, CLCN5, ALOX12 |

| **Supplemental table s2. Pathway analysis of predicted genes** | | |  |  |
| --- | --- | --- | --- | --- |
| **Term** | **Qvalue** | **Genes** | | |
| Pathways in cancer | 2.23E-07 | FGFR1, RET, FGF7, FGFR3, MAP2K1, FGF9, IGF1, EGLN1, FZD3, STAT1, FZD4, TGFB2, CCDC6, EDNRB, CCND1, CRKL, CDKN2B, VEGFA, RARB, FGF2, AKT3 | | |
| Cell cycle | 1.70E-04 | CDC6, CCND1, CDKN2B, CCND2, BUB1, CHEK1, YWHAE, CDC25A, WEE1, TGFB2 | | |
| Melanoma | 2.24E-04 | FGFR1, CCND1, FGF7, MAP2K1, FGF9, IGF1, FGF2, AKT3 | | |
| Proteoglycans in cancer | 2.55E-04 | FGFR1, CCND1, MAP2K1, CAMK2G, VEGFA, IGF1, HBEGF, FZD3, FGF2, FZD4, AKT3, TGFB2 | | |
| MicroRNAs in cancer | 3.18E-04 | NOTCH2, CCND1, CRKL, FGFR3, MAP2K1, CCND2, VEGFA, DICER1, BMPR2, BCL2L2, FZD3, PRKCE, CDC25A, TGFB2 | | |
| Signaling pathways regulating pluripotency of stem cells | 4.50E-04 | FGFR1, FGFR3, MAP2K1, BMPR2, IGF1, FZD3, FGF2, FZD4, ZFHX3, AKT3 | | |
| PI3K-Akt signaling pathway | 5.54E-04 | PPP2R1B, FGFR1, FGFR3, FGF7, MAP2K1, FGF9, IGF1, YWHAE, CCND1, CCND2, VEGFA, PRKAA1, MYB, FGF2, AKT3 | | |
| Ras signaling pathway | 7.87E-04 | FGFR1, FGFR3, FGF7, MAP2K1, ETS1, FGF9, ETS2, VEGFA, NF1, IGF1, FGF2, AKT3 | | |
| HTLV-I infection | 0.0024 | CCND1, CDKN2B, CCND2, ETS1, ETS2, CHEK1, TBP, FZD3, MYB, FZD4, AKT3, TGFB2 | | |
| Hippo signaling pathway | 0.0047 | PPP2R1B, CCND1, CCND2, BMPR2, FZD3, YAP1, FZD4, YWHAE, TGFB2 | | |
| Rap1 signaling pathway | 0.0096 | FGFR1, CRKL, FGFR3, FGF7, MAP2K1, FGF9, VEGFA, IGF1, FGF2, AKT3 | | |
| MAPK signaling pathway | 0.0097 | FGFR1, BDNF, CRKL, FGFR3, FGF7, MAP2K1, FGF9, NF1, FGF2, AKT3, TGFB2 | | |
| Pancreatic cancer | 0.0119 | CCND1, MAP2K1, VEGFA, STAT1, AKT3, TGFB2 | | |
| Renal cell carcinoma | 0.0119 | CRKL, MAP2K1, VEGFA, EGLN1, AKT3, TGFB2 | | |
| FoxO signaling pathway | 0.0121 | CCND1, CDKN2B, MAP2K1, CCND2, IGF1, PRKAA1, AKT3, TGFB2 | | |
| Bladder cancer | 0.0161 | CCND1, FGFR3, MAP2K1, VEGFA, HBEGF | | |
| Neurotrophin signaling pathway | 0.0345 | BDNF, CRKL, MAP2K1, CAMK2G, YWHAE, AKT3, ARHGDIA | | |
| AMPK signaling pathway | 0.0374 | PPP2R1B, CCND1, FASN, ELAVL1, IGF1, PRKAA1, AKT3 | | |
| Dorso-ventral axis formation | 0.0455 | NOTCH2, MAP2K1, ETS1, ETS2 | | |
| Thyroid cancer | 0.0557 | CCDC6, CCND1, RET, MAP2K1 | | |
